# Personal

**Published:** 1891-11

**Authors:** 


### Personal.

Dr. E. Arnold Praeger, of Nanaimo, B. C., read the address on Surgery at the recent meeting of the Canadian Medical Association. It was an able exposition of the present position of spinal surgery. Dr. Praeger is one of the most progressive and able of Canadian surgeons. He attended the annual meeting of the American Association of Obstetricians and Gynecologists in New York, and then went on to the Washington Congress of American Physicians and Surgeons. We trust that this genial citizen of British Columbia will visit the United States "early and often."

Dr. and Mrs. Henry Gibbons, Jr., of San Francisco, have started East on an eight months' vacation, and will soon sail for Europe. Most of this time will be spent in medical study and observation, and London will be mainly their residence while absent, where Dr. Gibbons, himself an experienced teacher, will have ample opportunity to compare and contrast English and American methods, especially with reference to obstetrics, which department he teaches in Cooper Medical College.

Dr. M. B. Ward, of Topeka, Kansas, accompanied by Mrs. Ward, visited the East, and read a paper before the American Association of Obstetricians and Gynecologists, at the New York Academy of Medicine, in September. Dr. Ward is one of the foremost physicians in the West, and his paper, entitled Removal of the Uterine Appendages, with Results, was full of interest, and was received and discussed with great enthusiasm.

Dr. John A. Ouchterlony, of Louisville, has presented to the Catholic University at Washington, D. C., a large and valuable archeological collection, illustrating the prehistoric antiquities of the Mississippi valley.—*Kansas Medical Journal.*

Dr. Edward Clark, of Buffalo, attended the annual meeting of the American Public Health Association, at Kansas City, October 19-23, 1891, where he read a paper on The Collection and Transportation of Garbage in Cities.

